# Rapid and conformal coating of polymer resins by airbrushing for continuous and high-speed roll-to-roll nanopatterning: parametric quality controls and extended applications

**DOI:** 10.1186/s40580-017-0105-2

**Published:** 2017-05-01

**Authors:** Jae Hyuk Lee, Minho Na, Jiyeop Kim, Kangeun Yoo, Jaekyu Park, Jeong Dae Kim, Dong Kyo Oh, Seungjo Lee, Hongseok Youn, Moon Kyu Kwak, Jong G. Ok

**Affiliations:** 1 Department of Mechanical and Automotive Engineering, Seoul National University of Science and Technology, Seoul, 01811 South Korea; 2 Department of Mechanical Engineering, Hanbat National University, 125 Dongseodaero, Yuseong-gu, Daejeon, 34158 South Korea; 3 School of Mechanical Engineering, Kyungpook National University, Daegu, 41566 South Korea

**Keywords:** Airbrushing, Thin film, Nanopattern, Roll-to-roll nanoimprint lithography, Residual layer thickness, Conformal coating

## Abstract

We present a facile and scalable coating method based on controlled airbrushing, which is suitable for conformal resin coating in continuous roll-to-roll (R2R) nanoimprint lithography (NIL) process. By controlling the concentration of UV-curable polymeric resin with mixing the volatile solvent and its airbrushing time, the coated resin film thickness can be readily tuned. After R2R NIL using a flexible nanoscale line pattern (nanograting) mold is conducted upon the airbrushed resin film, a large-area uniform nanograting pattern is fabricated with controlled residual layer thickness (RLT) based on the initial film thickness. We investigate the faithful airbrushing condition that can reliably create the uniform thin films as well as various nanopatterns with controlled morphologies. Using more diluted resin and shorter airbrushing time can reduce the RLTs favourably for many applications, yet is apt to induce the nanoscale pores and discontinued lines. We also discuss how to further improve the quality and scalability of resin airbrushing and its potential applications particularly requiring high-speed and conformal coating on highly topographic and flexible surfaces.

## Background

Roll-to-roll (R2R) nanoimprint lithography (NIL) enables large-area nanopatterning either on a rigid or flexible substrate by the continuous imprinting of the nanoscale pattern upon a polymeric resin film based on the pressurized rolling of a mold-bearing roll [1–4]. A wide choice of substrate materials, large processable area, and high speed of R2R NIL provide a unique methodology for the scalable and practical fabrication of many flexible and large-area applications ranging from optical and photonic components such as wire-grid polarizers [5, 6], metastructured optical filters [7], and antireflection films [8] through energy devices [9] to plasmonic sensors [10]. A successful R2R NIL is a combination of a well-prepared flexible mold that is large enough to wrap around the roll, a well-formulated resin that is suitable for high-speed R2R NIL with quick curing and smooth demolding, and a reliable resin coating method that can afford continuous R2R processing principle. While these three main aspects have been addressed in detail elsewhere [11], it still remains a challenge to establish a faithful resin coating process where typical spin-coating or drop-casting might obviously not be the best solution for coating a uniform resin film on a continuously feeding large-area substrate.

To this end, we present a facile, rapid, and scalable resin coating protocol by utilizing a controlled airbrushing technique. A UV-curable polymeric resin is diluted in a volatile solvent for smooth flowing, which is airbrushed over the substrate to leave a uniform thin resin film behind. This quick process is well-suited to the continuous and high-speed R2R NIL; we first review that a uniform nanoscale line (nanograting) pattern can be faithfully fabricated by conducting R2R NIL with a flexible nanograting mold onto the airbrushed resin films based on our previous work [12]. By modulating the resin concentration and airbrushing time, the initial thin film thickness as well as the residual layer thickness (RLT) of the nanopatterns created by R2R NIL can be readily controlled. We systematically investigate the parametric effects of the airbrushing process on the resulting nanopattern quality in our experimental space; reducing resin concentration and airbrushing time can lead to thinner RLTs usually favorable in most applications, yet is prone to cause the nanoscale pores and discontinued lines due to solvent evaporation and/or insufficient resin. We also discuss how to further improve the quality and scalability along with the potential applications that can benefit from the airbrushing and other extended large-area coating methodology such as the doctor blade coating.

## Results and discussion

### Process overview

A full experimental detail of airbrushing is described elsewhere [12]. Briefly, an airbrusher (compressed propellant blown at the maximum pressure of 0.15 MPa) connected to a compressed air cylinder and resist material vessel is mounted vertically over the substrate with the 15 cm distance. For the substrate materials, we choose polyethylene terephthalate (PET) films and Si wafers as the representatives of flexible and rigid substrates, respectively. In this study, we use epoxy-silsesquioxane (SSQ) mixed with 3 wt% photoacid generator as a UV-curable resist material [13] whose concentration can be modulated (e.g., 2, 5, 10, and 20 wt%) by diluting in propylene glycol methyl ether acetate (PGMEA). Airbrushing time of the diluted SSQ resist onto the target substrate is varied (e.g., 1, 2, and 3 s). A R2R NIL processing is performed on the SSQ-coated substrate by using the custom-built 400 mm-wide R2R NIL instrument [14], at the imprinting pressure of ~5 psi, followed by UV curing. Here, flexible molds bearing the 700 nm-period and 500 nm-deep nanograting and the microscale dot array are prepared by the soft lithography method [15] using polydimethylsiloxane (PDMS). The mold area can be scaled up as needed to wrap around the roll, by performing the tiling of master mold pieces in a slightly overlapped fashion [16].

**Fig. 1:**
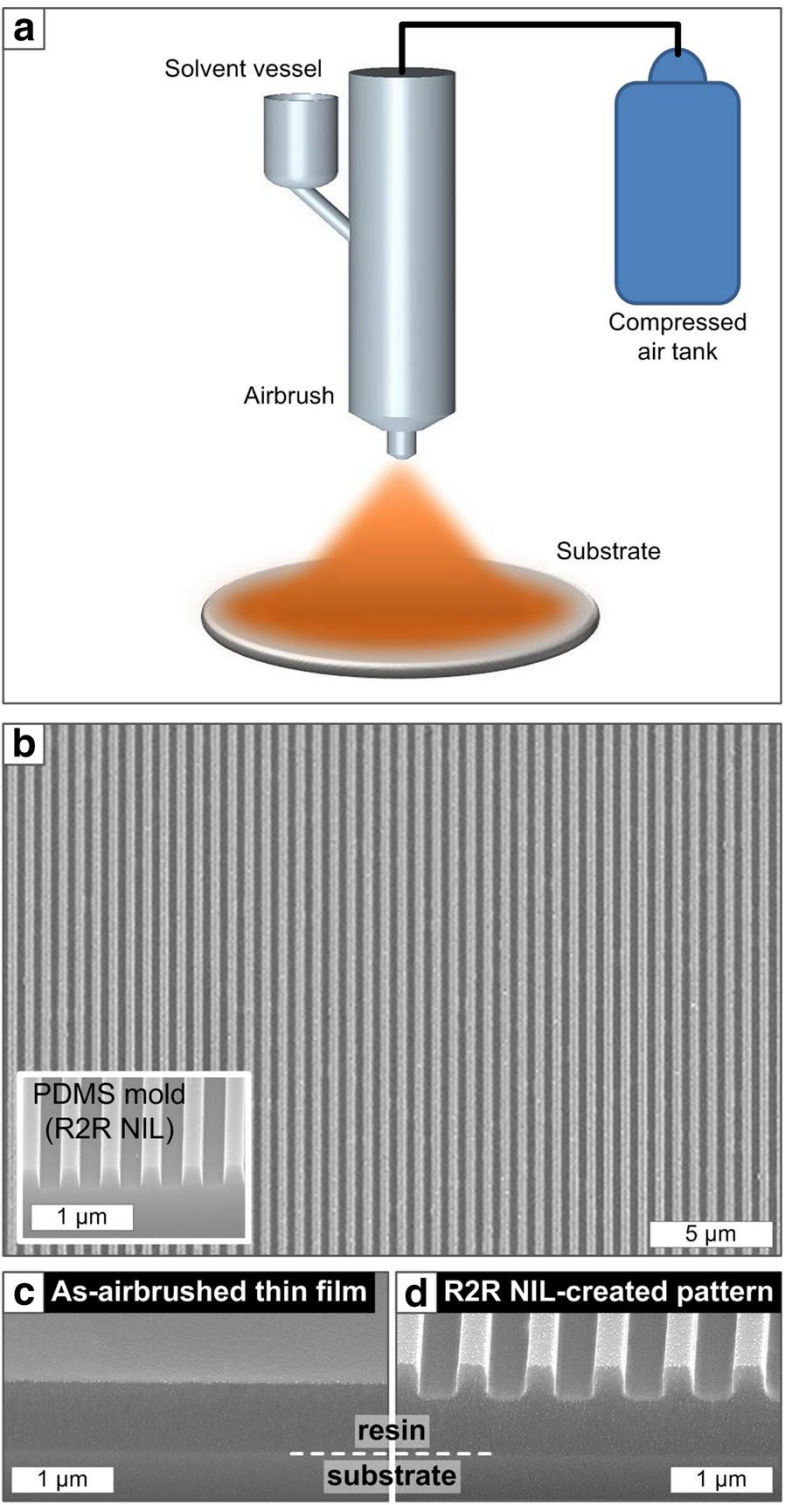
Schematics of airbrushing and exemplary results in R2R NIL. **a** Schematic illustration of an airbrushing process where the diluted polymer is airbrushed onto the substrate to form a uniform thin film. **b** SEM image (*top view*) of the nanograting pattern fabricated by performing R2R NIL on the UV-curable resin films airbrushed on a PET substrate. The *inset* to **b** shows the PDMS mold used in R2R NIL (45°-tilted SEM imaging). The 45°-tilted cross-sectional SEM images of **c** an initial thin film and **d** its nanoimprinted structure, obtained by airbrushing the 10 wt% SSQ on a Si substrate

Figure 1 shows the processing schematics (Fig. 1a) and the exemplary results of airbrushed thin SSQ resin films and R2R-nanoimprinted 700 nm-period nanograting patterns (Fig. 1b–d), where the 10 wt% SSQ resin was airbrushed for 2 s on the PET and Si substrates. The uniform thin SSQ film can be initially formed usually within 30 s as volatile PGMEA is rapidly dried off, which is suitable for R2R NIL that is dynamically conducted at high speed. Faithful results shown in Fig. 1b–d indicate that SSQ allows conformal deformation, rapid curing, and smooth demolding [13] in our R2R NIL on both PET and Si substrates. As previously investigated [12], the initial thin film thicknesses before NIL as well as the RLTs after NIL can be systematically tailored by controlling the resin concentration and airbrushing time; the thicker the SSQ and the longer the airbrushing time, the thicker the film thicknesses. Notably, this is valid as long as the resin concentration is within a proper range; too thick resin causes an uneven surface while too thin resin cannot form a continuous thin film after PGMEA is dried. In our case, an adequate concentration range of SSQ for making uniform thin films and later well-imprinted patterns is found to be between 5 and 10 wt% [12]. We focus on the two cases, 5 and 10 wt% with maximum propellant air pressure of 0.15 MPa, for parametric investigations hereafter, unless otherwise specified.

### Effect of resin concentration on nanopore generation

**Fig. 2:**
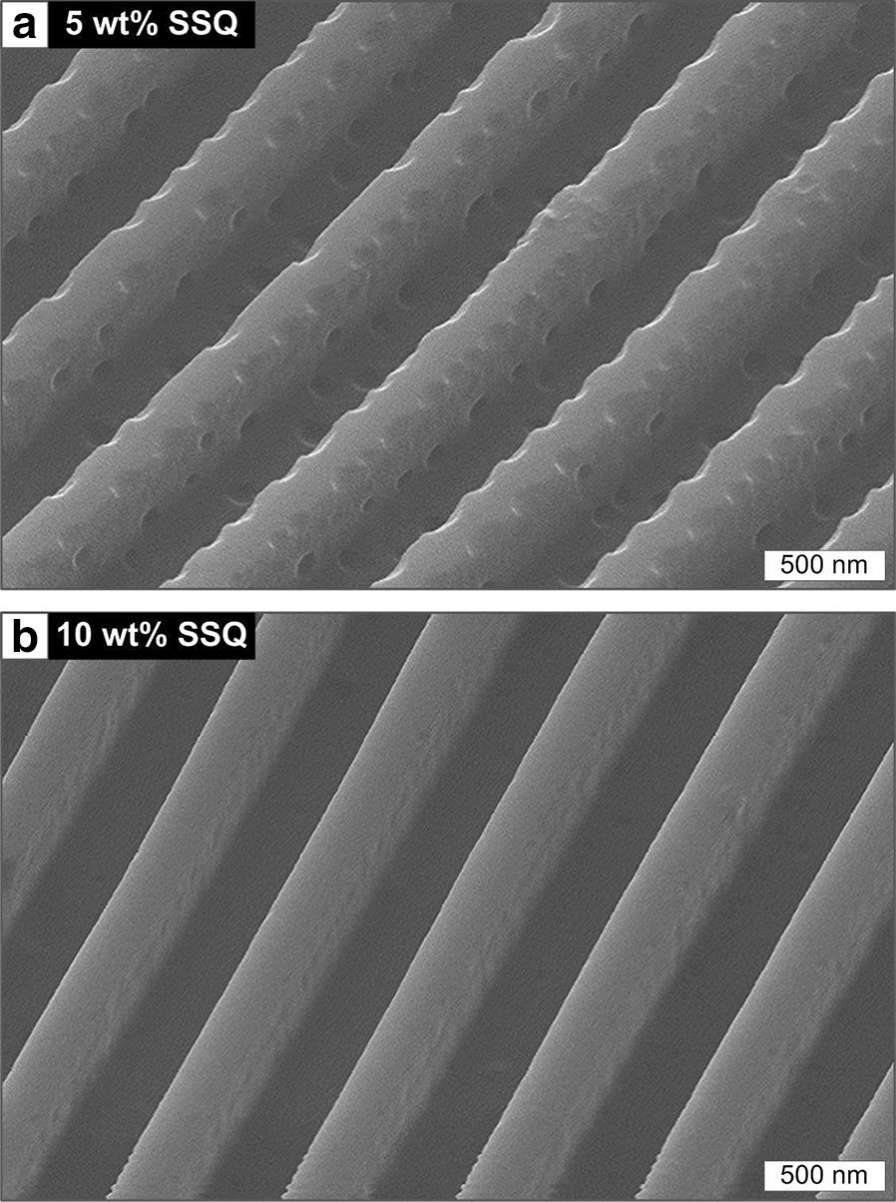
Morphology difference depending on the resin concentration: nanopore generation. SEM images of R2R NIL-fabricated 700 nm-period nanograting patterns on the airbrushed SSQ resin films with different concentrations of **a** 5 wt% and **b** 10 wt%. The nanoscale pores are generated when 5 wt% SSQ is airbrushed while not found in the 10 wt% SSQ case

Controlling RLTs as thin as possible during NIL is favorable as most of the following uses typically involve the RLT removal and/or require higher transmittance. In that sense, airbrushing of 5 wt% SSQ may overall be desirable without other issues. Figure 2, however, reveals another important perspective; use of 5 wt% SSQ generates nanoscale pores (Fig. 2a) while 10 wt% SSQ results in cleaner surfaces (Fig. 2b) in the imprinted nanopatterns. The nanopores cover both the top and side of nanograting structures as well as the background surfaces when 5 wt% SSQ is used (Fig. 2a). This is because the portion of volatile PGMEA in the apparently dried film is larger, which is outgassed to leave behind the pores while the non-volatile SSQ is crosslinked upon UV curing [17]. Not shown here, this phenomenon becomes more pronounced as the wt% of SSQ further decreases (e.g., 2 wt%), while it disappears once the SSQ concentration is larger than 10 wt% (Fig. 2b) in our experimental condition.

Though the nanopore generation may have an adverse influence for the normal purpose of obtaining smooth nanopattern surface, it can be useful for specific applications by utilizing its large surface area and/or rough topology. For instance, the nanopored nanostructures can make more advantageous templates for sensors and electrodes with increased surface areas, thereby enhancing the sensitivities and efficiencies. Additionally, the dimples in the nanopored surface can provide favorable spots for selective docking of the small particles such as cells [17], working as a functional nanoparticle sorting and trapping device [18].

### Effect of airbrushing time on nanograting continuity

**Fig. 3:**
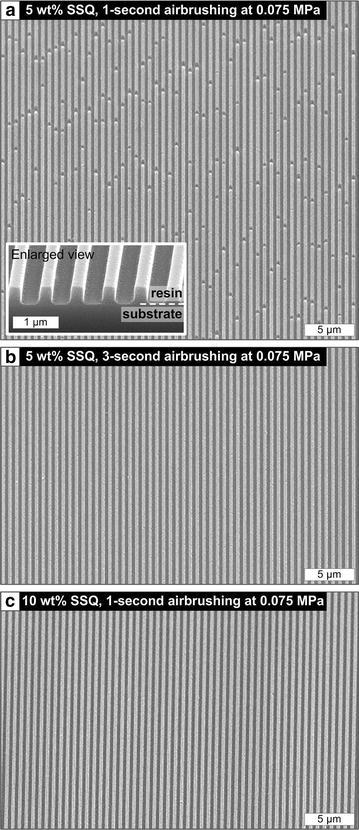
Morphology difference depending on the airbrushed resin amount: line continuity. SEM images of R2R NIL-fabricated 700 nm-period nanograting patterns on the airbrushed SSQ resin films at lowered pressure of 0.075 MPa with different concentrations and airbrushing times of **a** 5 wt% and 1 s, **b** 5 wt% and 3 s, and **c** 10 wt% and 1 s, respectively. The discontinued lines are observed in **a** as the insufficient resin dewets on the substrate surface upon UV curing. The *inset* to **a** shows an enlarged SEM image of the cross-section indicating a near-zero RLT. The increased amount of resin either by increasing the airbrushing time (**b**) or resin concentration (**c**) results in continuous nanograting under the identical R2R NIL condition

In the similar sense to the previous chapter, reducing the airbrushing time may be usually favorable for subsequent works with smaller RLTs. In our experimental space, a combination of shorter airbrushing time (e.g., 1 s), thinner SSQ (e.g., 5 wt%), and lowered propellant air pressures of 0.075 MPa, may result in the minimal, near-zero RLT (inset to Fig. 3a). However, Fig. 3a shows that such a condition is prone to produce the broken nanograting lines; the increase either in airbrushing time (Fig. 3b) or SSQ concentration (Fig. 3c) restores the results of continuous nanograting patterns. The line breakage observed in Fig. 3a is due to the insufficient amount of resin which undergoes dewetting during the curing process [19]. Such a fragmentized nanograting pattern can be of certain uses similar to the nanopored structures, for instance, multi-dimensional random patterns for backlight units in displays [20]. If the amount of resin in the initial thin film is sufficient enough to entirely fill up the openings in the nanograting mold upon the pressurized conformal contact, continuous nanograting pattern can be imprinted (Fig. 3b, c).

### Further applications and advances for conformal, scalable, and high-speed coating

Not only to the RLT-controllable resin coating for R2R NIL and simple planar thin film fabrication, can the airbrushing method also be applicable to more specific uses and broader materials. As the microscale droplets out of an airbrusher nozzle can smoothly reflow along the surface topography [12], airbrushing further enables the conformal coating over the surfaces especially with large surface roughness, which otherwise demands special treatments in ordinary spin coating or drop casting. By airbrushing, many functional materials can be conformally coated on highly topographic and flexible surfaces, including photoresists [21], anti-sticking agents [22], and active layers in flexible photovoltaic cells [23–25].

**Fig. 4:**
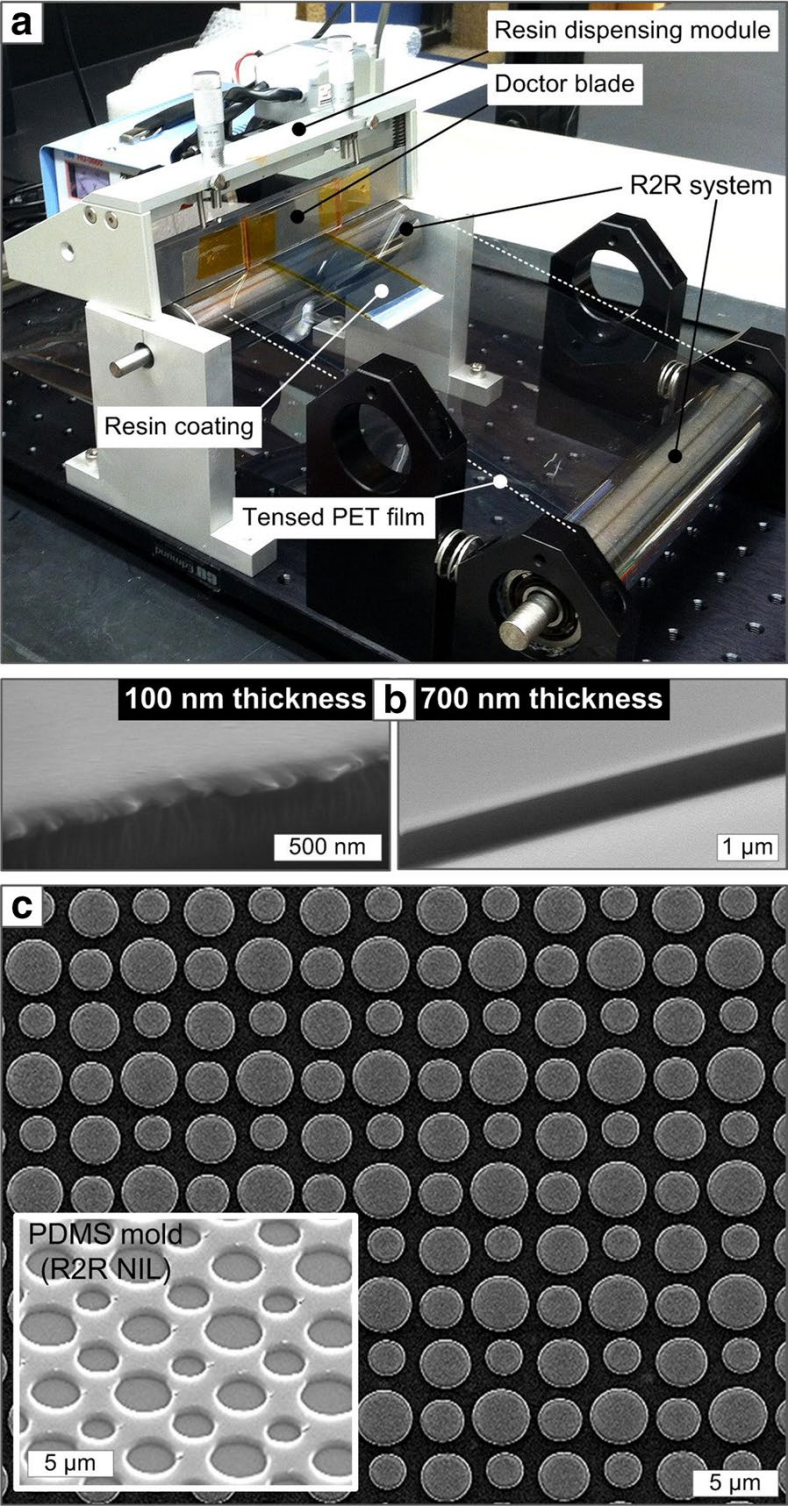
Doctor blade coating for rapid and scalable thin film fabrication and its nanopatterning application. **a** Optical image of a compact desktop doctor blade coating system equipped with a resin dispensing module and R2R conveyers. **b** Optical images of the doctor-bladed thin SSQ films on PET substrates. **c** SEM image of the microscale dot array fabricated on the doctor-bladed SSQ film by R2R NIL. The *inset* to **c** shows the PDMS mold used in R2R NIL (45°-tilted SEM imaging)

Moving forward, a controlled large-area conformal coating, particularly with a compatibility to continuous and high-speed processes like R2R NIL, can be realized by extended methodologies [26–28], at the head of doctor blade coating [29–31]; by uniformly spreading the controlled amount of materials through the small slits or gaps, target materials can be continuously coated over the large-area surfaces at high speed. Figure 4 demonstrates a compact doctor blade coater (Fig. 4a) along with an application example where the doctor blade method is used to coat the resin in R2R NIL for the fabrication of flexible metastructures comprising microscale dot array with various diameters (Fig. 4b, c) [7]. The film thickness can be controlled by modulating the doctor blading speed and the gap distance (Fig. 4b). Many diverse functional devices can benefit from the those methods to advance towards commercially-feasible scales, including light-emitting diodes [29, 30], transparent electrodes [32], photovoltaic cells [31, 33], and so forth.

## Conclusions

In summary, we introduce the airbrushing method for conformal and high-speed coating of functional polymers and demonstrate its application in continuous and scalable R2R NIL with controlled RLTs and further potential uses. The polymer film thickness as well as the surface morphology and profile can be modulated by regulating the concentration of UV-curable polymeric resin with mixing the volatile solvent and its airbrushing time. Using more diluted resin and shorter airbrushing time can reduce the RLTs favourably for many applications, while generating the surficial nanopores and/or dewetting-driven fragments upon UV curing. Airbrushing can be practically applied to many unique applications by enabling the conformal coating over the highly topographic and flexible surfaces at high speed, including but not limited to various functional coatings, electronics, and energy conversion devices.
